# Can a POCUS Clinical Decision Rule Improve Reliability in the Diagnosis of Paediatric Transient Synovitis of the Hip? A Single Centre Pilot Study

**DOI:** 10.24908/pocusj.v10i02.18290

**Published:** 2025-11-17

**Authors:** David J McCreary, Rashed Chowdhury, Cameron Hamilton

**Affiliations:** 1 Paediatric Emergency Department, Sunderland Royal Hospital, Kayll Road, Sunderland, GBR

**Keywords:** MSK POCUS, Joint effusion, Transient synovitis, Hip effusion, Clinical decision rule

## Abstract

**Objectives:**

*Primary*: To determine if point of care ultrasound (POCUS) combined with a clinical decision rule (CDR) improves reliability in the diagnosis of transient synovitis of the hip (TS) in paediatric patients presenting with atraumatic limp. *Secondary*: To describe how POCUS improves diagnostic reliability and reduces the need for further investigations for the child with atraumatic limp.

**Methods:**

We retrospectively applied a POCUS CDR to patients presenting to our paediatric emergency department (PED) with atraumatic limp over a 5-year period. This consisted of the following: ages 1 to 10 years old, able to weight bear, no history of fever, symptom duration for 7 days or less, and no pallor, lymphadenopathy, or hepatosplenomegaly.

**Results:**

A total of 77 out of 178 patients presenting to the PED with a diagnosis of TS underwent a POCUS examination during their clinical assessment. Of these, 67 patients had hip effusion on POCUS. Our CDR could be applied to correctly rule-in TS in 63 out of 67 patients. Ten patients did not have hip effusion; five of which were diagnosed with another cause for their limp and five were categorized as being possible TS. When POCUS was not utilised as part of clinical assessment, three cases included a misdiagnosis for children presenting with atraumatic limp.

**Conclusion:**

Our POCUS CDR could be applied to correctly rule-in TS in a very high proportion of cases. The integration of POCUS into the clinical assessment of children with atraumatic limp can reduce the need for unnecessary investigations while maintaining diagnostic reliability. We recognise that a large prospective study evaluating the role of a POCUS CDR is needed to further evaluate its reliability.

## Introduction

Atraumatic limp is a common reason for a child to present to the paediatric emergency department (PED) [1]. These patients can be challenging to diagnose due to the wide range of differential diagnoses underpinning their symptoms—including the more benign, such as transient synovitis of the hip (TS), to the much more serious, such as haematological malignancy [2]. TS, or “irritable hip,” represents the most common cause of atraumatic limp in a child with an incidence of 25.1 per 100,000 in those aged 0 to 14 years [3,4]. Most children with TS have a benign course and do not require extensive evaluation [5]. Distinguishing septic arthritis (SA) from TS can be difficult but important as both require considerably different management. The incidence of SA is considerably less than TS, estimated at 2 to 7 per 100,000 children [6]. The most well-known tool to differentiate TS from SA is the Kocher criteria [7].

Ultrasound is the most sensitive imaging modality for detecting synovial effusion compared to plain film radiographs and medical resonance imaging (MRI) scans and confers the advantages that it is quick, painless, and requires no sedation to achieve adequate imaging in paediatric patients [8]. Furthermore, there is no radiation risk to the patient [9]. Hip point of care ultrasound (POCUS) is relatively simple to learn and rapid to perform due to the prominent and readily identifiable sonographic landmarks [10]. Some limitations of POCUS include its operator dependence and the fact that the aetiology of the effusion cannot be discerned [11].

POCUS has been used in our department for the past five years to assess for hip joint effusion. During this time, we were able to demonstrate that when POCUS was utilised as part of the clinical assessment of children with atraumatic limp, a reduction in unnecessary blood tests and x-rays were achieved compared to when POCUS was not utilised. Following the work by Zoabi et al. in 2021 evaluating the use of POCUS as part of a decision support algorithm for suspected TS, we wanted to expand on the existing literature in this area and evaluate the reliability of our own POCUS clinical decision rule (CDR) for ruling-in TS [12].

## Methods

We retrospectively evaluated patients aged 1 to 16 years presenting with atraumatic limp over a 5-year period from May 2019 to May 2024. This included all admissions to the PED of our large District General Hospital with approximately 32,000 admissions per year. We included a total of 178 patients in our study who had a diagnosis of TS recorded at their initial visit. The patient demographic information is shown in Table 1. The electronic medical records of each patient were screened by all three authors who agreed on inclusion criteria. There were no disagreements between reviewers during this process. Information was collected on the gender and age of each patient along with the duration of limp or pain, weight-bearing status and whether the child had either self-reported fever during the symptomatic period or fever recorded at their PED visit. In addition to whether the child underwent a POCUS examination, we recorded if they received x-rays and blood tests. As a secondary objective we wanted to examine the effect that utilising POCUS had on the need for further investigations during the clinical assessment of children with atraumatic limp.

**Table 1.** Demographics and clinical features of patient cohort

**Gender**	
Male	120
Female	58
**Age**	
1-<3 years	67
3-<5 years	60
5-<7 years	28
7-<9 years	17
9-<11 years	3
11–16 years	3
**Preceding viral infection symptoms**	
Yes	86
No	92
**Duration of symptoms**	
<1	39
1-<4 days	97
4-<7 days	19
7+ days	23

**Figure 1.**
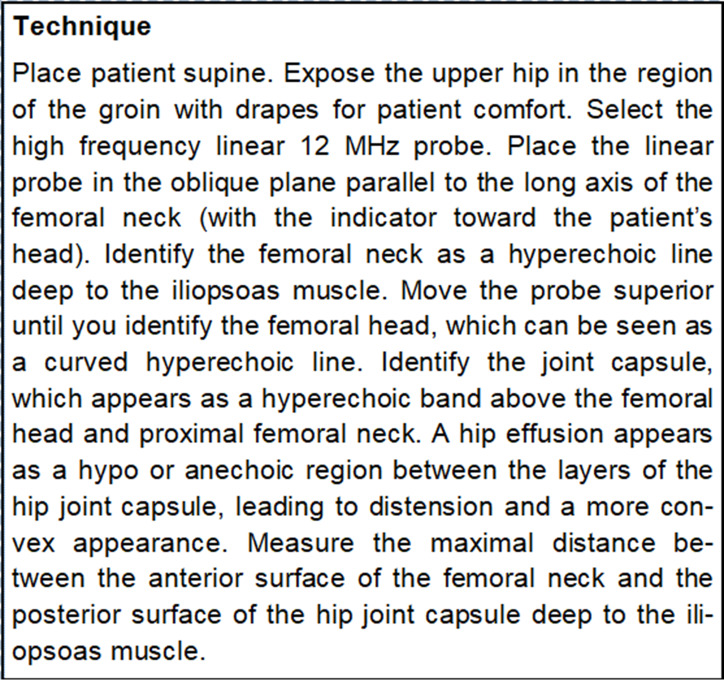
Technique for measuring hip effusion as defined in Paediatric Emergency Department Clinical Guideline: point of care ultrasound (POCUS) for the child presenting with atraumatic limp to the paediatric emergency department (PED)

**Figure 2.**
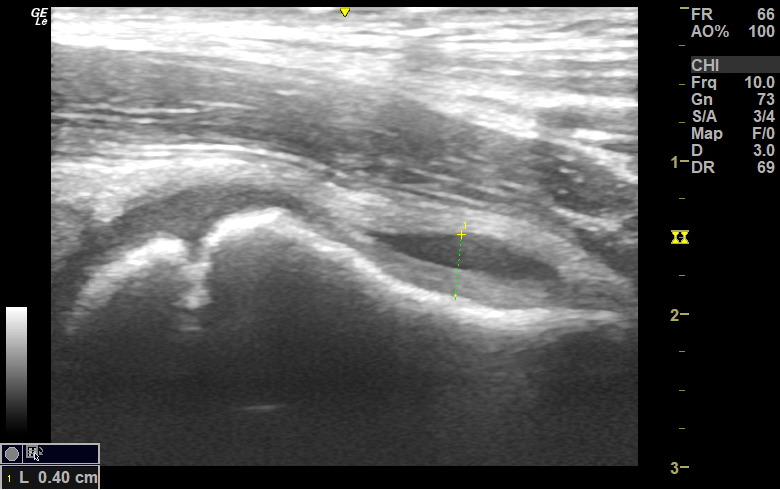
POCUS of hip demonstrating an effusion and how this was measured

As per existing departmental protocols for all patients with atraumatic limp follow-up was scheduled between 3 and 5 days after their initial presentation in order to ensure symptoms were improving. A diagnosis of TS was applied if the patient presented with limp or pain that was felt by the assessing clinician to be originating from the hip joint. This diagnosis was confirmed if the symptoms resolved at follow-up without any intervention other than oral anti-inflammatory medications, or if there was no re-attendance with similar symptoms in the event of failure to attend follow-up. In this retrospective sample, scans were conducted if there were clinicians who had undergone specific training on paediatric POCUS hip scanning available. Training consisted of a full day face-to-face paediatric-specific POCUS course that covered upper and lower musculoskeletal scanning modalities. This was delivered through our Sunderland Ultrasound Society (SUNUS) training course led consultants (senior physicians) in paediatric emergency medicine, all of whom held specific postgraduate qualifications in paediatric POCUS. Operators were deemed competent to perform the scans after directly supervising at least 15 paediatric hip scans by their mentors. All scans were carried out using specified departmental protocols (see Figure 1) and were conducted using the GE (Boston, MA, USA) LOGIQ ultrasound system using the 12 MHz high frequency linear probe. All images obtained were reviewed by one of two consultants who were ultrasound trainers within the SUNUS faculty (as described above). The size of the hip effusion was also recorded (See Figure 2). As described in Figure 2, the measurement was taken from the anterior surface of the bony cortex to the posterior aspect of the joint capsule and was deemed significant if it represented a difference of more than 0.2 cm compared to the unaffected hip. In rare cases of bilateral effusion, a measurement greater than 0.5 cm in size was deemed significant, consistent with existing practice [13,14].

Scanning technique for identifying hip effusion as adapted from Sunderland Royal Hospital Paediatric Emergency Department Clinical Guideline*: point of care ultrasound (POCUS) for the child presenting with atraumatic limp to the paediatric emergency department (PED)*

Our CDR consisted of the following components: age 1 to 10 years, able to weight bear, afebrile, symptom duration for 7 days or less, and no features of pallor, lymphadenopathy, or hepatosplenomegaly. All components of this CDR had to be met in order for it to be applied.

We felt our CDR was appropriate for this pilot study. It mirrored that produced by Zoabi et al. but with an amended age range to include children aged 1 to10 years, rather than ages 3 to 10 years, which we believe would exclude a significant proportion of young children for whom a diagnosis of TS can be reliably made [12]. This has been supported by previous service evaluations within our department following the introduction of POCUS protocols for hip scanning over the past 5 years.

This retrospective study was approved by the South Tyneside and Sunderland NHS Foundation Trust Research and Ethics Committee.

## Results

We included 178 patients in our study. The male to female ratio was 1.4:1. A total of 77 out of 178 patients (43%) underwent POCUS as part of their assessment. Of these, 67 patients showed a hip effusion using POCUS, including 66 unilateral and 1 bilateral. The mean size of hip effusion in our study was 0.53 cm. For those with hip effusion on POCUS, our CDR could only be applied if all five criteria were met. Using these criteria, we were able to correctly rule-in TS in 63 out of 67 patients. In the remaining four cases, four of the five criteria of our CDR were met. One patient had a symptom duration of greater than 7 days; two patients were not weight bearing at presentation but then were fully weight bearing at follow-up; and one patient self-reported fever during the period of limp. All four of these patients underwent blood tests as per our departmental Limping Child Pathway Protocol (See Supplemental File 1). This includes undertaking a full blood count and inflammatory markers and blood culture if sufficient clinical concern exists. In each of these cases, the white cell count and C-reactive protein/erythrocyte sedimentation rate were normal.

Out of 77 patients who underwent POCUS examination as part of their clinical assessment, 10 did not have evidence of hip effusion. For 5 of the 10 patients in this group, their symptoms had resolved at follow-up and they were diagnosed as being “possible TS.” We acknowledged that during the early stages of TS, hip effusion may not be evident or may be very small and difficult to detect. It is entirely likely that an alternate cause such as a minor soft tissue injury was the cause of symptoms in this group. All patients in this group were less than 3 years of age and had a symptom duration of less than 48 hours with full resolution at follow-up. One of these scans were described as “difficult to obtain” (due to the child not cooperating) and therefore may have provided evidence of effusion if the scan was repeated under other circumstances. Accounting for these factors suggests that our CDR would have performed even better than we have reported.

In the remaining five patients with no hip effusion on their scans, a negative POCUS prompted the clinician to seek alternative diagnoses other than TS. In three of these cases a diagnosis was reached either during the initial assessment or during follow-up with orthopaedic and rheumatology specialty teams. In two cases, symptoms were ongoing, and formal diagnoses had not yet been reached (See Table 1).

Significantly, within our cohort of patients who did not undergo POCUS imaging as part of their assessment there were three misdiagnoses of TS, where an alternate, correct diagnosis was later identified at follow-up. These patients and their final diagnoses are outlined in Table 2. We believe that if POCUS had been part of the clinical assessment of these children, the absence of hip effusion would have prompted the clinicians assessing them to consider an alternate diagnosis much like in the cases of those with a negative POCUS exam.

**Table 2.** Patients with no hip effusion identified on POCUS and TS excluded with eventual diagnoses

Gender	Age	Duration of limp (at presentation)	Other investigations	Eventual diagnosis
F	3 years	3 days	Bloods MRI head	Post infectious cerebellar ataxia
F	7 years	1 days	Bloods NAD x-rays NAD Repeat ultrasounds large joints normal	Unclear, currently receiving rheumatology follow up, juvenile idiopathic arthritis (JIA) excluded
M	5 years	4 days	x-rays normal bloods NAD	Non-inflammatory joint pain associated with hypermobility
F	1 year	1 day	x-rays normal bloods NAD	Unclear, intermittent limp, currently receiving follow up from orthopaedic team
F	2 years	2 days	x-rays normal bloods NAD	Unclear, currently receiving follow up from orthopaedic team and physiotherapy input

Out of 178 patients in our study, 21 (11%) did not attend follow-up at 3-5 days. However, the advice given to carers at our institution is that if the symptoms are resolving, it is reasonable to contact the follow-up clinic to cancel their appointment. Within this group of 21 patients there were no re-attendances with the same symptoms within 30 days. Therefore, we believe it is reasonable to assume no clinically significant cases were missed and all diagnoses of TS made were accurate ones.

In the 77 children who underwent POCUS, 10 (12.9%) underwent blood tests. Of these, three had symptom durations of 7 days, two had large effusions (greater than 0.8 cm), and one had fever. The remaining four patients did not have effusions on POCUS. This is comparative to the group where POCUS was not used, in which 37 out of 101 children (36.6%) underwent blood tests.

Only 11 out of 67 (16.4%) who had hip effusion on POCUS received x-rays of the pelvis. All were justifiable and in keeping with departmental protocols based on their age or symptom duration of greater than 7 days. Three patients without effusion on POCUS had x-rays and all went on to receive other eventual diagnoses (see Table 3). In comparison, in the group of 101 patients who did not undergo POCUS, 43 (42.5%) had x-rays of the pelvis. In this group, no cases of bony pathology were identified on x-ray. This demonstrates that the use of POCUS led to fewer unnecessary x-rays in over 25% of cases.

**Table 3.** Patients mis-diagnosed as TS without using POCUS and their eventual diagnoses

Gender	Age	Duration of limp	Eventual diagnosis
M	1.5 years	Unclear	Tip toe walking felt to be consistent with autism spectrum disorder, later diagnosed
M	5 years	2 days	Brodies abscess left femur identified on MRI
M	1.5 years	3 days	Toddlers fracture, identified on x-ray at 48 hour follow up

Apart from the POCUS imaging, no formal radiology-performed ultrasounds were carried out in our cohort. While there are paediatric radiologists available to undertake ultrasound scans for hip effusion in our department, this service is not widespread. As a result, most scans are undertaken by those working in the PED who have undertaken sufficient training and who are deemed competent to do so.

## Discussion

Clinical prediction algorithms or decision rules have been described as valuable tools for distinguishing between SA and TS in children in terms of improving diagnostic accuracy and for providing a structured approach to clinical evaluation [15].

Our primary objective was to evaluate how a POCUS CDR can be used to improve reliability in the diagnosis of TS. We wanted to build on the work by Zoabi et al. who utilised a POCUS CDR for ruling-in TS in children with atraumatic limp. Their study demonstrated a high sensitivity and positive predictive value of a similar CDR. We found that our POCUS CDR had a very high degree of reliability in correctly identifying TS in 63 out of 67 patients (94%) with hip effusion on POCUS. When POCUS was utilised, there were no cases of misdiagnosis of TS (i.e., false positives, compared with the three cases occurring in the non-POCUS group). In contrast, for the five patients where POCUS did not reveal hip effusion, other alternate diagnoses were sought by the clinician and were correctly identified either acutely or during follow-up. We feel this highlights the importance of POCUS and the advantage it can confer. POCUS can help a clinician to rule-in TS, rather than it being solely considered a diagnosis of exclusion, which is how it has traditionally been described [16]. To our knowledge, our study is novel as it is the only example of a POCUS CDR for atraumatic limp being evaluated in a population in the United Kingdom. Importantly, within our cohort there were no cases of SA—reflecting its low incidence [17].

One male patient in our study, aged 7 years, had symptoms for more than 14 days and was found to have bilateral hip effusions on POCUS, both at presentation and at follow-up. MRI scans identified equivocal findings for Perthes disease. However, 15 months after his original presentation, this diagnosis was yet to be confirmed during orthopaedic follow-up, despite repeat MRI. There is some evidence that Perthes disease sufferers may present with hip joint effusion early in their presentation [18,19]. This echoes the findings of Zoabi et al., in which two male patients, both aged 10 years, were later diagnosed with Perthes disease after presenting with hip effusions on their scans. The patient in our study was 7 years old and thus underwent x-rays of his pelvis as part of his initial assessment, as per our Limping Child Pathway Protocol. These initial x-rays were reported as normal, and therefore may not have been helpful at this point in his presentation.

Another novel feature of our study was our secondary objective, where we investigated the impact of POCUS in terms of avoiding unnecessary x-rays and blood tests for a child during their clinical assessment. There is sufficient supporting evidence that for children aged 10 years and younger with limp, no fever, and less than one week's onset, can have a clinical diagnosis of TS made without extensive investigations [20,21]. We have demonstrated that by using POCUS, the number of children who undergo x-rays and venepuncture can be significantly reduced. This can reduce pain and distress for patients and their families and reduce the patient's exposure to unnecessary radiation.

Limitations of this study include the limited number of patients who underwent POCUS. This was due to a lack of trained clinicians being available when patients presented to the PED. This highlights the need for more clinicians to be trained in the use of musculoskeletal POCUS, something which has been demonstrated both in the United Kingdom and in the United States of America [22,23,24]. Another limitation is the retrospective nature of the application of this CDR. A study in which a POCUS CDR is prospectively applied would limit any potential recall bias and produce results which are more reliable. A further limitation of the study relates to the lack of an objective reference standard for the diagnosis of TS. In our study, a diagnosis of TS was applied if the patient presented with limp and pain which was felt by the assessing clinician to be originating from the hip joint, which then resolved at the following attendance without any intervention other than oral anti-inflammatory medications and one where no re-attendance with similar symptoms occurred. Although these criteria provide a degree of robustness and reflect the criteria used for TS in clinical practice, they do not represent a definitive diagnosis. A final limitation related to the operator-dependent nature of POCUS. Although all scans were performed according to a departmental protocol and overseen by clinicians with extensive experience in hip scanning, a degree of bias could have been introduced.

## Conclusion

Integrating POCUS into the clinical assessment of children presenting with atraumatic limp is quick, safe and was well tolerated by patients in our study. Our objective was to evaluate the reliability of POCUS as part of a CDR in correctly identifying TS. In this pilot study, our POCUS CDR could be applied to correctly rule-in TS in a very high proportion of cases. We believe this pilot study provides evidence that integrating POCUS into clinical assessment and diagnosis for children with atraumatic limp can reduce the need for unnecessary blood tests and x-rays, while maintaining diagnostic reliability. We recognise that there is a requirement for a large prospective study evaluating the role of a POCUS CDR in order to further evaluate its reliability.
