# Supplementary material for: Can a POCUS Clinical Decision Rule Improve Reliability in the Diagnosis of Paediatric Transient Synovitis of the Hip? A Single Centre Pilot Study
Source: POCUS J. 2025 Nov 17;10(2):103–8. doi: 10.24908/pocusj.v10i02.18290 (PMC12658581; doi:10.24908/pocusj.v10i02.18290)
Supplement: Supplementary file 1 [file pocusj-10-02-18290-s001.pdf]

South Tyneside and Sunderland
NHS Foundation Trust

Clinical Guideline

Title	**The Limping Child Pathway**
Directorate	Paediatrics
Reference	CG0866
Author(s)	Lorna Gillespie, Charlotte Atkinson, Raj Khanna, Chris McKie, Sue Gray
Ratifying Group	Paediatrics and Child Health Clinical Governance Group
Clinical Director Approval	Dr Lorna Gillespie
Date of Ratification	13/07/2021
Date of Implementation	04/08/2021
Date for Review	01/12/2025

Guideline Statement	South Tyneside Paediatric Pathways
Applies to / Target group	All staff in South Tyneside and Sunderland NHS Foundation Trust who provide Care within the Urgent Care for Children Service

Version History Log

Version	Release	Author	Details of significant changes
1.0		Charlotte Atkinson	New document
2.0	Dec 2022	Charlotte Atkinson	Reviewed document

It is the responsibility of all users of these pathways to ensure that the correct versions are being used.

Document Status: This is a controlled document. Whilst this document may be printed, the electronic version posted on the intranet is the controlled copy. Any printed copies of this document are not controlled. As a controlled document, this document should not be saved onto local or network drives but should always be accessed from the intranet.

Clinical Pathway: The Limping Child

Definition: Limp means an asymmetric gait that deviates from the usual age-appropriate gait pattern, usually caused by pain, weakness or deformity.

General Points: Limp is a clinical presentation, not a diagnosis. Limping may be due to pain referred from somewhere else (genital / spinal pain referring to the hip, and pain from the hip referring to the thigh or knee). Safeguarding should always be considered.

Exclusions: This pathway **does not** apply to children with a **definite history of trauma**.

Initial Assessment: Perform a full set of observations including pulse, respiratory rate, temperature, and oxygen saturations, and calculate PEWS. Ensure appropriate analgesia is given.

History:

- Duration and progression of limping
- History or possibility of trauma
- Any precipitating factors
- Pattern of severity and pain
- Nocturnal pain and symptoms
- Functional limitations (e.g. complete inability to weight bear)
- Any associated muscle weakness / change in bladder or bowel habit
- Systemic symptoms: fever, night sweats, rigors, rash
- Constitutional Symptoms e.g. weight loss, lethargy, anorexia
- Recent viral illness
- Birth, developmental and family history.

Examination:

- **Generalised** including fever, tachycardia, pallor, Abdomen, lymph nodes, scrotum, petichiae / pupura
- **pGALS** (paediatric Gait, Arms, Legs, Spine)
- **Joint examination** using 'Look, Feel and Move' including joints above and below the area of pain
    - Look: resting limb position, leg length disparity, swelling, deformities, skin changes e.g. rash, wounds, bruising
    - Feel: heat, cold, tenderness, crepitus, fluctuance
    - Move: active, passive, compare both sides, tone, power and reflexes
- **Neurovascular** assessment of affected limb

CAUSES OF LIMP BY AGE		
**LESS THAN 3 YEARS**	**3 – 10 YEARS**	**OLDER THAN 10 YEARS**
Septic Arthritis<br><br>Osteomyelitis<br><br>Fracture / Soft Tissue Injury<br><br>Developmental Dysplasia of the Hip<br><br>Toddler Fracture<br><br>Acute Myositis<br><br>Non-Accidental Injury<br><br>Transient Synovitis of the Hip (less common below 3 years)	Transient Synovitis<br><br>Septic Arthritis<br><br>Osteomyelitis<br><br>Fracture / Soft Tissue Injury<br><br>Perthes Disease<br><br>Acute Myositis	Septic Arthritis<br><br>Osteomyelitis<br><br>Slipped Upper Femoral Epiphysis<br><br>Perthes Disease<br><br>Fracture / Soft Tissue Injury<br><br>Osgood Schlatter's Disease<br><br>Severs Disease<br><br>Osteochondritis Dissecans<br><br>Chondromalacia Patellae
**ANY AGE**		
Septic Arthritis / Osteomyelitis<br>Malignancy including leukaemia<br>Non-malignant haematological disease e.g. haemophilia, sickle cell<br>Metabolic Disease e.g. Rickets<br>Neuromuscular Disease e.g. cerebral palsy, spina bifida<br>Primary Limb abnormality e.g. length discrepancy<br>Inflammatory joint or muscle disease e.g. JIA, Lyme Arthritis<br>Non-musculoskeletal conditions including intra-abdominal pathology e.g. appendicitis, inguinal hernia, and inguinoscrotal disorders e.g. testicular torsion		

- ✓ Symptoms for less than 72 hours, or greater than 72 hours and improving.
- ✓ Mobile but limping
- ✓ Well
- ✓ No red flags

- Symptoms for more than 72 hours and no improvement
- No red flags

INFECTION (SA / OM) Red Flags

- Temperature >38.5 in preceding week
- Unable to weight bear
- Pain on moving joint (passive)

MALIGNANCY Red Flags

- Fatigue, anorexia, weight loss, night sweats
- Pain waking child at night

Investigations:

No investigations indicated if all of the following apply:

- No red flags in history or examination
- Ambulating with mild or no discomfort with simple analgesia
- Clear working diagnosis and / or plan for follow up.

Imaging:

- X-Ray (area of suspicion)
- If hip pain with red flags then perform pelvis XR for hip pain (AP if over 4 years old, AP and Frog Leg Lateral if over 8 years old)
- Ring 42135 and ask for PEM consultant to request assistance with XR interpretation if needed
- Ultrasound can be used as an adjunct to diagnosis in terms of confirming presence of a hip effusion, but it should be noted that it does not differentiate between causes of effusion and does not exclude bony pathology e.g. Perthes

Laboratory:

- FBC, CRP and ESR; Blood cultures if pyrexial

Amended Kocher's Criteria for Septic Arthritis (SA):

Fever >38.5C; Unable to weight bear; CRP >20mg/L; WCC >12

1 criteria = 3% probability for SA; 2 criteria = 40%; 3 criteria = 93%; 4 criteria = 99.6%

Treatment:

- Definitive management is determined by the working diagnosis
- Simple analgesia and child-directed limitation of activity suitable for the majority of cases.
- If in doubt, discuss with the duty PED consultant at Sunderland Royal, extension 42135

Referral / Disposal:

Orthopaedic FU / Virtual Trauma Clinic: Confirmed fracture / confirmed toddler fracture

ST Rapid Access Clinic: Non-traumatic persistent limp with no red flags or normal investigations; if wait >72 hours consider referral to SRH PEM Trauma clinic instead

Orthopaedic Referral: Septic Arthritis (orthopaedic emergency, consider discussion direct with SRH Orthopaedic Registrar), Osteomyelitis, Perthes Disease, SUFE

Paediatric Consultant Referral / Advice: Suspected NAI; Suspected malignancy on investigations, suspected neuromuscular disease
